# HIV-hepatitis co-infection in a rural community in Northern Nigeria

**DOI:** 10.11604/pamj.2020.36.352.23978

**Published:** 2020-08-27

**Authors:** Oluwaseyitan Andrew Adesegun, Olabiyi Hezekiah Olaniran, Emmanuel Bamidele, Joseph Nicholas Inyang, Michael Adegbe, Tolulope Oyinloluwa Binuyo, Osaze Ehioghae, Oluwafunmilola Adeyemi, Oyekunle Oyebisi, Akolade Olukorede Idowu, Oluwafemi Ajose

**Affiliations:** 1 General Hospital, Garaku, Nasarawa State, Nigeria,; 2 Benjamin Carson (Snr.) School of Medicine, Babcock University, Ilishan-Remo, Ogun State, Nigeria,; 3 Federal Medical Centre, Keffi, Nasarawa State, Nigeria,; 4 Department of Community Medicine, Babcock University Teaching Hospital, Ilishan-Remo, Ogun State, Nigeria,; 5 Oyo State Ministry of Health, Ibadan, Oyo State, Nigeria,; 6 College of Medicine, University of Ibadan, Ibadan, Oyo State, Nigeria,; 7 Department of Internal Medicine, Olabisi Onabanjo University Teaching Hospital, Sagamu, Ogun State, Nigeria,; 8 Department of Internal Medicine, Babcock University Teaching Hopital, Ilishan-Remo, Ogun State, Nigeria,; 9 School of Public Health, University of South Wales, Wales, United Kingdom

**Keywords:** Public health, HIV/AIDS, hepatitis B, hepatitis C, rural medicine, Nigeria, Africa

## Abstract

**Introduction:**

HIV, hepatitis B and hepatitis C pose a public health challenge in sub-Saharan Africa and there are only few studies on co-infection of these viruses done in rural areas in Northern Nigeria. This study provides a rural perspective on HIV-hepatitis co-infection in a Northern Nigerian community.

**Methods:**

this cross-sectional study was carried out amongst people living with HIV/AIDS (PLWHA) in a rural community hospital over a three-month period. Socio-demographic data and other relevant information were obtained from the participants and case notes using an interviewer-administered questionnaire. Hepatitis B surface antigen and antibody to Hepatitis C virus were assayed from serum using enzyme-linked immunosorbent assay (ELISA) kits developed by LabACON®. Chi-square test was used to compare categorical variables and logistic regression modelling was used to determine correlates of co-infection in the population.

**Results:**

a total of 281 individuals participated in the study. The prevalence of Hepatitis B co-infection, Hepatitis C co-infection and triple infection was 6.0%, 14.6% and 1.1% respectively. Using Chi-square test, none of the socio-demographic characteristics, WHO Clinical Stage, viral suppression had significant association with Hepatitis B co-infection, however marital status was significantly associated with Hepatitis C co-infection and level of education was significantly associated with triple infection (p < 0.05). Logistic regression modelling generated no significant results.

**Conclusion:**

co-infection of viral hepatitis (particularly Hepatitis C) in PLWHA is common in rural Northern Nigeria, and significant correlates include lack of formal education and being married. There is need for provider-initiated routine counselling and screening of PLWHA for viral hepatitis, with adequate follow-up and treatment of co-infected individuals and Hepatitis B vaccination for those without co-infection.

## Introduction

Human Immunodeficiency Virus (HIV) infection and chronic viral hepatitis (Hepatitis B and C virus infection) are global health problems that are of concern particularly to the African people, as the prevalence of these chronic illnesses remain high in the continent, particularly the sub-Saharan region [1-3]. About 37 million individuals are infected with HIV globally, out of which 5-20% are also co-infected with Hepatitis B virus (HBV) [4]. Chronic viral hepatitis affects about 70 million people in Africa (60 million with Hepatitis B and 10 million with Hepatitis C) [5]. Co-infection of any of these hepatotropic viruses with HIV hastens the progression of liver disease in these patients [6,7].

Nigeria, a country with a population of about 200 million people is known to have the second highest prevalence of HIV/AIDS in the world, recently pegged at 1.4% in 2019, an improvement from previous years [8]. The prevalence of HBV infection in Nigeria is between 12.2-14%, while the prevalence of Hepatitis C virus (HCV) infection ranges from 2.8 to 24.2% amongst adults from different sub-regions of the country [9-12]. Nasarawa State, a State in the north-central part of the country is home to many viruses, as high prevalence rates of HIV, Hepatitis B and Hepatitis C have been documented in the State. A recent update by the National Agency for the Control of AIDS (NACA) placed the State in the top ten list of States with the highest prevalence of HIV in Nigeria, with prevalence rate of 1.9%. Prevalence of Hepatitis B in Nasarawa State ranges between 7.1% and 13.3% [13-15], while the prevalence of Hepatitis C has been quoted at around 13.2% to 24.2% [9,15]. Co-infection of one or more of the Hepatitis viruses with HIV is not uncommon, as these viruses all share similar routes of transmission including unsafe sexual practices, mother-to-child transmission, unsafe blood transfusion, injection with contaminated sharps in the healthcare setting, traditional tattooing and scarification to mention a few. Recent studies in the State have shown HIV-HBV co-infection rates to be about 11%, while HIV-HCV co-infection rates were about 13.5% and 5% of the HIV positive individuals had both HBV and HCV in a particular study [14]. This implies a higher risk of progression of liver disease to liver cirrhosis and hepatocellular carcinoma amongst co-infected individuals [16,17].

Studies providing information on the state of HIV-hepatitis co-infection in rural Nigeria, including the current screening practices for these viruses are lacking. This study will fill a gap in knowledge on this subject, providing information relevant for a region with high prevalence rates of HIV, Hepatitis B and Hepatitis C. This study will also inform hospital practices and policies as regards serological screening practices in HIV positive individuals who present to the hospital for medical care. The objectives of this study were to determine the prevalence of Hepatitis-Hepatitis co-infection (HIV-Hepatitis B co-infection, Hepatitis C co-infection and HIV-Hepatitis B-Hepatitis C co-infection / Triple infection), and ascertain factors associated with HIV-Hepatitis co-infection, as well as correlates of HIV-Hepatitis co-infection among people living with HIV/AIDS (PLWHA) in a Northern Nigerian community.

## Methods

**Study area**: the study was carried out in a secondary healthcare centre located in Garaku (General Hospital, Garaku), the local government headquarters of Kokona Local Government Area in Nasarawa State. This hospital, which is the only secondary healthcare facility serving the area, provides healthcare to indigenes of the town, as well as other smaller villages in the local government. The hospital provides care to PLWHA in collaboration with the Institute of Human Virology Nigeria (IHVN), Garaku, by running an anti-retroviral therapy (ART) clinic, providing HIV Voluntary Counselling and Testing (VCT), Provider-initiated testing, treatment and follow-up, and management of complications.

**Study population**: the target population for this study was all HIV positive individuals who presented to the ART clinic during the study period, who were not known to have Hepatitis B or Hepatitis C, and had not been screened for Hepatitis B and Hepatitis C in the last 6 months. All those who had been previously diagnosed with Hepatitis B or Hepatitis C, or who had been screened for any of two viruses within the last 6 months were excluded.

**Study design**: the study was a descriptive cross-sectional study, carried out over a three-month period, from December 2019 to February 2020. Data was obtained from the participants and from their case notes using an interviewer-administered questionnaire, which was applied by trained research assistants. The age, gender, occupation (grouped according to the International Standard Classification of Occupations) [18], level of education, marital status, viral suppression (HIV RNA <1000 copies per millilitre) [19] and current WHO clinical stage were obtained and documented. Blood samples (5 millilitres) were obtained by venepuncture from the antecubital fossa and centrifuged to separate serum from packed cells. Hepatitis B surface antigen (HBSAg) and antibody to Hepatitis C virus (anti-HCV) were assayed using enzyme-linked immunosorbent assay (ELISA) kits developed by LabACON® (Hangzhou Biotest Biotech Co., Ltd, China). The manufacturer´s instructions on the use of the kits were strictly adhered to.

**Sample size determination**: the minimum sample size was determined using the Kish formula (n = Z^2^pq/d^2^, where n = sample size, p = prevalence, q = 1-p, d = absolute precision) [20] to be approximately 260, after finite population correction, assuming a confidence level of 95% and 5% absolute precision, with a prevalence of 28.4% from a previous study [21].

**Sampling technique**: a multistage sampling technique was employed. The sample size (260) was divided into three strata, based on the month of th ART clinic appointments for the clients (December, January, February), with at least 87 participants allotted to each stratum. Since each month (stratum) had 8 clinic days on average, at least 11 participants were randomly selected on each clinic day throughout the study period, until the minimum sample size was reached.

**Data collection and analysis**: data was entered into the IBM Statistical Package for Social Sciences (SPSS) Version 22 (Chicago, IL, USA) and summary statistics were generated. Chi-square test was used to compare categorical variables, while univariate/multivariate logistic regression was used to model the relationship between socio-demographic variables and HIV-Hepatitis co-infection in the population. Unadjusted odds ratio (OR) and adjusted odds ratio (aOR) were generated for the univariate and multivariate logistic regression models respectively, and their associated 95% confidence intervals computed. Age, gender, level of education, occupation, marital status, WHO clinical stage and viral suppression were used as socio-demographic (independent) variables, while HIV-Hepatitis B co-infection, HIV-Hepatitis C co-infection and HIV-Hepatitis B-Hepatitis C co-infection (triple infection) each served as the dependent variables. Level of significance was set at p < 0.05.

**Ethical considerations**: ethical approval was obtained from the ethics review committee of the Federal Medical Centre, Keffi, Nasarawa State, Nigeria, with registration number NHREC/21/12/2012. Written informed consent was obtained from study participants.

## Results

**Socio-demographic characteristics of respond ents**: a total of 281 respondents were sampled, 79.7% of whom were female while 20.3% were male. The mean age of respondents was 37.1±9.5, with the age of respondents ranging between 7 and 68 years. Most respondents (43.8%) were however within the 31-40 age group (Table 1, Table 1 (suite)). Majority of the participants (43.4%) had no prior formal education, whereas 23.5% had primary, 25.3% had secondary and 7.8% had tertiary education. Two hundred and forty four (86.8%) of the respondents were married, while 24 (8.5%) and 13 (4.6%) respondents were single and divorced/separated respectively. The occupations of the respondents are classified in line with the International Standard Classification of Occupations (Table 1, Table 1 (suite)).

**Table 1:** socio-demographic characteristics of study participants

Variable		N=275	Percentage (%)
Age group	0-10	1	0.4
	11-20	5	1.8
	21-30	63	22.4
	31-40	123	43.8
	41-50	62	22.1
	51-60	23	8.2
	61-70	4	1.4
Gender	Male	57	20.3
	Female	224	79.7
Level of education	None	122	43.4
	Primary	66	23.5
	Secondary	71	25.3
	Tertiary	22	7.8
Marital status	Single	24	8.5
	Married	244	86.8
	Divorced/Separated	13	4.6

**Table 1(suite):** socio-demographic characteristics of study participants

Category	Frequency	Percentage (%)
**Skilled agricultural, forestry and fishery workers**		
Subsistence farmers	176	62.6
**Service and Sales workers**		
Food vendors	3	1.1
Hair dressers	8	2.8
Petrol vendors	2	0.7
Petty traders	31	11.0
**Professionals**		
Teachers	8	2.8
Health worker	1	0.4
Banker	1	0.4
**Craft and related trade workers**		
Soap producer	8	2.8
Miller	1	0.4
Mason	1	0.4
Tailor	8	2.8
**Technician and associate professional**		
Health volunteer	1	0.4
**Plant and machine operators at assemblies**		
Drivers	2	0.7
Civil Servants †	8	2.8
Unemployed	11	3.9
Students §	11	3.9
**TOTAL**	281	100

† Civil Servants - refers to those who reported their occupation to be “civil servant”, without specifying their role in the civil service. This designation is not part of the International Standard Classification of Occupations. Unemployed - This designation is not part of the International Standard Classification of Occupations. § Students - This designation is not part of the International Standard Classification of Occupations

**HIV status and co-infection with viral hepatitis**: all of the respondents had been previously diagnosed with HIV and commenced on Highly Active Anti-Retroviral Therapy (HAART). Two hundred and seventy five (97.9%) of the study participants were in WHO clinical stage 1, while 5 (1.8%) were in stage 2 and 1 (0.4%) in stage 3. Two hundred and fifty participants (89%) had achieved HIV viral suppression (defined as viral load <1000 copies per ml) [19], while 11% had not achieved HIV viral suppression, as documented in their case notes. However, only 224 (80.4%) of the respondents had their most recent viral load tests done within the last 12 months; 54 (19.2%) had their viral load checked within the last 24 months while 1 participant´s result was from the last 36 months. Seventeen participants (6.0%) had Hepatitis B co-infection, while 41 participants (14.6%) had Hepatitis C co-infection. Three individuals (1.1%) had dual co-infection with Hepatitis B and Hepatitis C (triple infection). Our study also showed that majority of those who had Hepatitis B co-infection, Hepatitis C co-infection and triple infection were between 31 and 40 years of age, female, skilled agricultural workers, though the occupation of those with triple infection was evenly distributed between civil servants, professionals and the unemployed (Table 2, Table 2 (suite), Table 2 (suite 1)). While most of the respondents who had Hepatitis B and C co-infection were uneducated, two-third of those with triple infection had tertiary education. Furthermore, majority of the respondents with Hepatitis B co-infection, Hepatitis C co-infection and triple infection were married, in WHO clinical stage 1 and had achieved HIV viral suppression (Table 2, Table 2 (suite), Table 2 (suite 1)).

**Table 2:** association between socio-demographics and co-infection

Variable		Hep B co-infection		p-value	Hep C co-infection		p-value	Triple infection		p-value
		Pos (%)	Neg (%)		Pos (%)	Neg (%)		Pos (%)	Neg (%)	
**Age**	0-10	0 (0.0)	1 (0.4)	0.901	0 (0.0)	1 (0.4)	0.599	0 (0.0)	1 (0.4)	0.544†
	11-20	0 (0.0)	5 (1.9)		0 (0.0)	5 (2.1)		0 (0.0)	5 (1.8)	
	21-30	3 (17.6)	60 (22.7)		7 (17.1)	56 (23.3)		0 (0.0)	63 (22.7)	
	31-40	9 (52.9)	114 (43.2)		22 (53.7)	101 (42.1)		3 (100)	120 (43.2)	
	41-50	3 (17.6)	59 (22.3)		7 (17.1)	55 (22.9)		0 (0.0)	62 (22.3)	
	51-60	2 (11.8)	21 (8.0)		4 (9.8)	19 (7.9)		0 (0.0)	23 (8.3)	
	61-70	0 (0.0)	4 (1.5)		1 (2.4)	3 (1.3)		0 (0.0)	4 (1.4)	
**Gender**	Male	6 (35.3)	51 (19.3)	0.123	9 (22.0)	48 (20.0)	0.834	1 (33.3)	56 (20.1)	0.495
	Female	11 (64.7)	213 (80.7)		32 (78.0)	192 (80.0)		2 (66.7)	222 (79.9)	

† - Likelihood ratio Chi-square used in place of Pearson Chi-square when assumptions for contingency tables larger than 2 x 2 were violated. - Fisher's exact test used when the assumptions for a 2x2 contingency table was violated. Level of significance (p-value) < 0.05

**Table 2(suite):** association between socio-demographics and co-infection

Variable		Hep B co-infection		p-value	Hep C co-infection		p-value	Triple infection		p-value
		Pos (%)	Neg (%)		Pos (%)	Neg (%)		Pos (%)	Neg (%)	
Occupation	Civil Servant	2 (11.8)	6 (2.3)	0.113†	1 (2.4)	7 (2.9)	0.651†	1 (33.3)	7 (2.5)	0.082†
	Craft	2 (11.8)	16 (6.1)		2 (4.9)	16 (6.7)		0 (0.0)	18 (6.5)	
	Plant and Machinery Operators	0 (0.0)	2 (0.8)		0 (0.0)	2 (0.8)		0 (0.0)	2 (0.7)	
	Professionals	1 (5.9)	9 (3.4)		1 (2.4)	9 (3.8)		1 (33.3)	9 (3.2)	
	Service and Sales	1 (5.9)	43 (16.3)		6 (14.6)	38 (15.8)		0 (0.0)	44 (15.8)	
	Skilled agricultural workers	9 (52.9)	167 (63.3)		30 (73.2)	146 (60.8)		0 (0.0)	176 (63.3)	
	Students	0 (0.0)	11 (4.2)		0 (0.0)	11 (4.6)		0 (0.0)	11 (4.0)	
	Technicians	1 (5.9)	0 (0.0)		0 (0.0)	1 (0.4)		0 (0.0)	1 (0.4)	
	Unemployed	1 (5.9)	10 (3.8)		1 (2.4)	10 (4.2)		1 (33.3)	10 (3.6)	

† - Likelihood ratio Chi-square used in place of Pearson Chi-square when assumptions for contingency tables larger than 2 x 2 were violated. Level of significance (p-value) < 0.05

**Table 2(suite 1):** association between socio-demographics and co-infection

Variable		Hep B co-infection		p-value	Hep C co-infection		p-value	Triple infection		p-value
Pos (%)	Neg (%)		Pos (%)	Neg (%)		Pos (%)	Neg (%)	
Level of Education	None	9 (52.9)	113 (42.8)	0.269†	19 (46.3)	103 (42.9)	0.350	0 (0.0)	122 (43.9)	**0.026†**
	Primary	3 (17.6)	63 (23.9)		13 (31.7)	53 (22.1)		0 (0.0)	66 (23.7)	
	Secondary	2 (11.8)	69 (26.1)		7 (17.1)	64 (26.7)		1 (33.3)	70 (25.2)	
	Tertiary	3 (17.6)	19 (7.2)		2 (4.9)	20 (8.3)		2 (66.7)	20 (7.2)	
Marital Status	Single	0 (0.0)	24 (9.1)	0.206†	0 (0.0)	24 (10.0)	**0.012†**	0 (0.0)	24 (8.6)	0.653†
	Married	16 (94.1)	228 (86.4)		40 (97.6)	204 (85.0)		3 (100)	241 (86.7)	
	Divorced/Separated	1 (5.9)	12 (4.5)		1 (2.4)	12 (5.0)		0 (0.0)	13 (4.7)	
WHO Clinical Stage	Stage 1	17 (100)	258 (97.7)	0.685†	40 (97.6)	235 (97.9)	0.809†	3 (100)	272 (97.8)	0.937†
	Stage 2	0 (0.0)	5 (1.9)		1 (2.4)	4 (1.7)		0 (0.0)	5 (1.8)	
	Stage 3	0 (0.0)	1 (0.4)		0 (0.0)	1 (0.4)		0 (0.0)	1 (0.4)	
Viral Suppression	Suppressed	16 (94.1)	234 (88.6)	0.704	37 (90.2)	213 (88.8)	1.000	3 (100)	247 (88.8)	1.000
	Unsuppressed	1 (5.9)	30 (11.4)		4 (9.8)	27 (11.3)		0 (0.0)	31 (11.2)	

† - Likelihood ratio Chi-square used in place of Pearson Chi-square when assumptions for contingency tables larger than 2 x 2 were violated; - Fisher's exact test used when the assumptions for a 2x2 contingency table was violated. Level of significance (p-value) < 0.05

There was no statistically significant association between the socio-demographic characteristics, WHO Clinical Stage, HIV viral suppression and Hepatitis B co-infection using Chi-square test (p > 0.05) (Table 2, Table 2 (suite), Table 2 (suite 1)). However, marital status was significantly associated with Hepatitis C co-infection (p < 0.05), as 97.6% of those who had Hepatitis C co-infection were married (Table 2, Table 2 (suite), Table 2 (suite 1)). Other socio-demographic characteristics, along with WHO clinical stage and HIV viral suppression had no association with Hepatitis C co-infection (p > 0.05) (Table 2, Table 2 (suite), Table 2 (suite 1)). Only level of education had a significant association with HIV-Hepatitis B-Hepatitis C (triple) infection (p < 0.05), amongst all the variables tested (Table 2, Table 2 (suite), Table 2 (suite 1)) with two-third (66.7%) of those that had triple infection having tertiary education (Table 2, Table 2 (suite), Table 2 (suite 1)). Results of logistic regression analyses for the HIV-Hepatitis B co-infection model, after systematic removal of variables with extremely high or low odds ratio (unadjusted and adjusted odds ratios) indicated that the generated models were no different from null models, suggesting that none of the independent variables were sufficiently correlated with HIV-Hepatitis B co-infection. A similar finding was obtained when HIV-Hepatitis C and Triple infection were set as the dependent variable.

## Discussion

The goal of the study was to establish the prevalence and factors associated with Hepatitis co-infection in a rural population of PLWHA in Nigeria. It was found that most of the study participants were aged 31-40 years. This finding is in agreement with recent national statistics that indicate that the burden of HIV infection in Nigeria is highest in individuals aged 15- 49 years (about 75% of all PLWHA) [22]. More women (randomly selected) were found to have participated in this study, which may suggest that more women attended the ART clinic during the study period. This is generally in keeping with national statistics that reports over half of PLWHA in Nigeria to be females, in both rural and urban areas [22-24]. A study conducted in Keffi, a town not far from the study location also yielded similar male-female distribution, while another report from a study conducted six years prior, in the same hospital, confirmed the pattern [14,25]. On another note, this finding may also suggest a higher clinic attendance and health-seeking behaviour in women, though this was not an objective of the study and hence was not properly assessed. The above referenced report by the National Agency for the Control of AIDS (NACA) also shows that females with HIV had a higher incidence of sexually transmitted infections, a report which is in tandem with our findings, which showed that females had a proportionately higher prevalence of Hepatitis B co-infection, Hepatitis C co-infection and HIV-Hepatitis B-Hepatitis C triple infection [22,24].

Our study shows that more women had Hepatitis B, Hepatitis C and both co-existing with HIV, which is similar to previous studies conducted in the same region of Northern Nigeria[14,25]. However, studies in South-east and Southwest Nigeria found males to be more likely to have co-infection with the Hepatitis viruses [26,27]. Our report also shows that the burden of HIV-Hepatitis co-infection is higher in middle aged individuals than in any other age bracket, especially in those aged 31-40 years, similar to what was found six years prior in the same study population [25]. The prevalence of Hepatitis B co-infection, Hepatitis C co-infection and triple infection was 6%, 14.6% and 1.1% respectively. This prevalence rate for Hepatitis B co-infection is much less than the 13% reported in the same community (Garaku) years ago, and the other higher rates reported in the State, as well as the national average [13-15, 25]. The story is however different for Hepatitis C co-infection rates which are notably higher than Hepatitis B co-infection both in our study and the above mentioned Keffi study [14]. Varying co-infection rates have also been reported in different parts of the country, from 1.9% in Ekiti and 3% in Ibadan, to 23.5% in Abeokuta [27-29]. Prevalence of viral hepatitis is generally noted to be comparatively higher in Northern Nigeria than the Sothern parts of the country [30]. Education and occupation could be likely drivers of this problem, as most of our respondents who had co-infection (particularly Hepatitis B and Hepatitis C) were uneducated agricultural workers, though most of those who had triple infection had tertiary education. This finding of education affecting co-infection in general is not alarming, as the study was carried out in a rural community where lack of formal education is commonplace. It is possible that a lack of formal education could preclude some individuals from utilizing their clinic visits to the utmost, by obtaining all needed information from the healthcare givers. It is also possible for a lack of formal education to favour poor health-seeking behaviours. This hence suggests the need for healthcare workers, particularly those caring for PLWHA in rural settings to give voluntary counselling and screening for Hepatitis B and Hepatitis C to PLWHA.

Worthy of note is the fact that a large majority of our respondents with co-infection were married. This suggests the perpetuation of co-infection within married couples, and possible transmission to their offspring, more so in a culture that permits polygamy. This assumption is however subject to confounding by other variables such as the presence or absence of extramarital sexual relations, one partner being a chronic carrier prior to marriage and other known risk factors for HIV, Hepatitis B and Hepatitis C infection. This finding however raises questions on why there are more married PLWHA who are co-infected. Interestingly, most of our respondents were in WHO clinical stage 1 and had achieved viral suppression (< 1000 RNA copies/ml), an acceptable cut-off in the developing world. This was a noteworthy observation, and is indicative of efforts of the caregivers in encouraging drug compliance, as well as the patients´ willingness to adhere to the anti-retroviral medications. It is however not yet common practice for healthcare providers to request routine viral hepatitis screening for PLWHA in Nigeria. A similar study in Jos however found that most of their respondents who had Hepatitis B co-infection and triple infection had higher HIV RNA loads and had more severe immunosuppression prior to the initiation of HAART [31]. Some of the limitations of this study include the fact that we did not evaluate the presence of risk factors (modes of transmission) for hepatitis B and Hepatitis C, though this may have aided in the establishment of some associations. We were also unable to run other viral markers such as Hepatitis B core antigen, which would have provided further details on the serostatus of the participants, as it relates to chronic carriage and infectivity. This was due to the limited resources available for the study.

## Conclusion

This study found a higher Hepatitis C co-infection rate relative to Hepatitis B co-infection rate, and a low rate of triple infection. Lack of formal education, as well as being married were significant correlates of triple infection and HIV-Hepatitis C co-infection respectively, from this study. The authors recommend that healthcare providers for PLWHA be aware of the risk of their patients contracting Hepatitis B and Hepatitis C, particularly those living in the Northern part of Nigeria where there is a high prevalence of HIV, Hepatitis B and Hepatitis C, and hence provide annual or biannual routine voluntary counselling and screening for them, to possibly correspond with the routine viral load checks.

### What is known about this topic

- It is known that HIV, Hepatitis B and Hepatitis C are prevalent in sub-Saharan Africa, more so in Nigeria, and co-infection of these viruses in an individual is inevitable.

### What this study adds

- This study gives a snapshot of the situation in a rural community in Northern Nigeria, where the prevalence of HIV, Hepatitis B and Hepatitis C are known to be high;
- It gives the prevalence rates of co-infection for an entire local government and also identifies significant correlates of Hepatitis B and Hepatitis C co-infection amongst PLWHA in a rural community which include lack of formal education and marital status (being married).

## References

[1] World Health Organization (2006). HIV/AIDS, WHO, Regional Office for Africa, World Health Organization.

[2] World Health Organization (2019). Hepatitis B: World heal. Organ Fact Sheet. World Health Organization.

[3] Mercy Jelagat Karoney, Abraham Mogisi Siika (2013). Hepatitis C virus (HCV) infection in Africa: a review. Pan Afr Med J.

[4] (2016). Joint United Nations Program on HIV/AIDS. Global AIDS Update.

[5] World Health Organization (2019). Hepatitis, WHO, Regional Office for Africa. World Health Organization.

[6] Irena Maier, George Wu Y (2002). Hepatitis C and HIV co-infection: a review. World J Gastroenterol, Baishideng Publishing Group Inc.

[7] Kasha P Singh, Megan Crane, Jennifer Audsley, Anchalee Avihingsanon, Joe Sasadeusz, Sharon R Lewin (2017). HIV-hepatitis B virus coinfection: epidemiology, pathogenesis, and treatment. AIDS, NIH Public Access.

[8] Federal Ministry of Health, UNAIDS, National Agency for the Control of AIDS (NACA) (2019). New survey results indicate that Nigeria has an HIV prevalence of 1.4%. World Health Organization.

[9] Pennap G, Nuhu I, Oti V (2016). Prevalence of hepatitis C virus infection among people attending a voluntary screening centre in Masaka, Nasarawa State, Nigeria. Asia J Appl Microbiol.

[10] Ahinge Godwin I, Malu Abraham O, Mbaave Peter T, Bitto Terkaa T, Shaahu Vivian N, Mohammed Hameed (2013). Prevalence of hepatitis C in Makurdi, North Central Nigeria. IOSR J Dent Med Sci.

[11] Victor Omote, Emmanuel Kashibu, Isreal Ojumah, Danjuma Adda, Johnson Etaghene, Henry Ukwamedua (2018). Serological screening of hepatitis B virus and hepatitis C virus among patients attending a tertiary hospital in Jalingo, Taraba state, Nigeria. Saudi J Heal Sci.

[12] Adebola Olayinka T, Akin Oyemakinde, Muhammad Balogun S, Anthonia Ajudua, Patrick Nguku, Moses Aderinola (2016). Seroprevalence of Hepatitis B infection in Nigeria: a national survey. Am J Trop Med Hyg American Society of Tropical Medicine and Hygiene;.

[13] Alisigwe OJ (2019). Seroprevalence of hepatitis B virus infection among the students of Nasarawa State University, Keffi, Nigeria. Glob Sci J.

[14] Okwori A, Alabi S, Ngwai Y, Makut M, Obiekezie S, Ishaleku D (2013). The Seroprevalence of Hepatitis B and C Virus Co-Infection among HIV-1-infected patients in Keffi, North Central Nigeria. IOSR J Dent Med Sci.

[15] Pennap GR, Yakubu A, Oyige O, Forbi J (2010). Prevalence of hepatitis B and C virus infection among people of a local community in Keffi, Nigeria. African J Microbiol Res.

[16] Rosenthal E, Roussillon C, Salmon-Céron D, Georget A, Hénard S, Huleux T (2015). Liver-related deaths in HIV-infected patients between 1995 and 2010 in France: the Mortavic 2010 study in collaboration with the Agence Nationale de Recherche sur le SIDA (ANRS) EN 20 Mortalité 2010 survey. HIV Med.

[17] Chun HM, Mesner O, Thio CL, Bebu I, Macalino G, Agan BK (2014). HIV outcomes in Hepatitis B virus coinfected individuals on HAART. J Acquir Immune Defic Syndr, NIH Public Access.

[18] International Labour Organization (2012). International Standard Classification of Occupations. Geneva.

[19] Consolidated guidelines on the use of antiretroviral drugs for treating and preventing HIV infection - second edition (2016). World Health Organization.

[20] Kish L (1965). Survey Sampling: New York: John Wiley and Sons, Inc.

[21] Taiwo Modupe Balogun, Samuel Emmanuel, Emmanuel Folorunso Ojerinde (2012). HIV, Hepatitis B and C viruses´ coinfection among patients in a Nigerian tertiary hospital. Pan Afr Med J.

[22] (2017). National Agency for Control of AIDS (NACA), Federal Ministry of Health, Natl Strateg Framew. HIV AIDS 2017.

[23] National Agency for the Control of AIDS (NACA) (2019). FACT SHEET: HIV Prevention program. National Agency for the Control of AIDS (NACA).

[24] National Agency for the Control of AIDS (NACA) National HIV/AIDS epidemilogy and impact analysis (NHEIA) Report.

[25] Gyar S, Agbo P, Reuben C (2014). Assessment of Hepatitis B Co-infection among HIV/AIDS patients attending antiretroviral therapy (ART) clinic in Garaku, Central Nigeria. Res J Microbiol.

[26] Okocha E, Okonkwo U, Asomugha L, Odenigbo C, Oguejiofor O (2012). Prevalence of hepatitis B surface antigen seropositivity among HIV-infected and non-infected individuals in Nnewi, Nigeria. Niger Med J Medknow.

[27] Jesse Otegbayo A, Babafemi Taiwo O, Titilola Akingbola S, Georgina Odaibo N, Kayode Adedapo S, Sudhir Penugonda (2008). Prevalence of hepatitis B and C seropositivity in a Nigerian cohort of HIV-infected patients, Ann Hepatol. Elsevier.

[28] Adeolu Sunday Oluremi, Oluyinka Oladele Opaleye, Bosede Abimbola Babalola, Mujeeb Shittu, Oladipo Abayomi (2014). Coinfection of Hepatitis B and C virus among HIV patients visiting specialist hospital in Ikole Ekiti, Nigeria. J Med Sci Clin Res.

[29] Sandra Olukemi Ogwu-Richard, David Ajiboye Ojo, Olusola Abiodun Akingbade, Iheanyi Omezuruike Okonko (2015). Triple positivity of HBsAg, anti-HCV antibody, and HIV and their influence on CD4+ lymphocyte levels in the highly HIV infected population of Abeokuta, Nigeria. Afr Health Sci, Makerere University, Medical School.

[30] Umego C, Mboto C, Mbim E, Edet U, George U, Tarh J (2018). Epidemiology of Hepatitis B virus infection in South-South, Nigeria: a review Int STD Res Rev.

[31] Nimzing Gwamzhi Ladep, Patricia Aladi Agaba, Oche Agbaji, Auwal Muazu, Placid Ugoagwu, Godwin Imade (2013). Rates and impact of hepatitis on human immunodeficiency virus infection in a large African cohort. World J Gastroenterol Baishideng Publishing Group Inc.

